# Dietary patterns of Chinese women of childbearing age during pregnancy and their relationship to the neonatal birth weight

**DOI:** 10.1186/s12937-020-00607-y

**Published:** 2020-08-26

**Authors:** Hui Yan, Shaonong Dang, Yaodong Zhang, Shuying Luo

**Affiliations:** 1 The Children’s Hospital Affiliaten to Zhengzhou University; Henan Children’s Hospital, Zhengzhou Children’s Hospital, Zhengzhou, 450018 China; 2 School of Public Health, Xi’an Jiaotong University, Xi’an, 710061 China

**Keywords:** Pregnant women, Dietary pattern, Birth weight, Cross-sectional studies

## Abstract

**Objective:**

To examine the type of maternal dietary patterns during pregnancy and the distribution characteristics of children’s birth weight and the association between dietary patterns and neonatal birth weight in China.

**Methods:**

Data were derived from a cross-sectional program named “The prevalence and risk factors of birth defects in Shaanxi Province” in July to November in 2013. A stratified multistage random sampling method was used to select women and their children. The mother’s diet during pregnancy was investigated using semi-quantitative food frequency questionnaire (FFQ) to collect the frequency and amount of food consumption, and the newborn birth weight as well as related social demographic information was collected at the same time. In our study, 0–1 year old children and their mothers with complete dietary survey data were selected as research objects. The main dietary patterns were identified according to factor analysis, and latent class analysis (LCA) was used to investigate the social demographic factors affecting dietary patterns. The logistic regression model was used to assess the association between birth weight and maternal dietary patterns during pregnancy by establishing three adjusting models and the data were stratified for further analysis by urban-rural and regions.

**Results:**

A total of 15,980 participants were involved in this study. Four dietary patterns were identified: “vegetarian pattern”, “balance pattern”, “traditional pattern” and “processing pattern”. Compared with moderate tertile, women in the highest tertile of adherence to vegetarian pattern increased the risk of low birth weight in offspring in rural areas (*OR* = 1.61, 95%*CI:*1.06–2.93) and middle region (*OR* = 1.75, 95%*CI*:1.18–2.62), and the traditional pattern had greater odds of lower birth weight in the middle region (*OR* = 1.55, 95%*CI*:1.05–3.75). The processing pattern was found a protective factor for the occurrence of low birth weight in rural areas (*OR* = 0.98, 95%*CI*:0.43–0.99) but was a risk factor for low birth weight in the southern region (*OR* = 8.83, 95%*CI*:1.22–15.16). The balance pattern was a protective factor for the occurrence of low birth weight in the northern region(*OR* = 0.35, 95%*CI*:0.14–0.83).

**Conclusion:**

The vegetarian and traditional pattern may be positively related to a higher risk of low birth weight while the balanced pattern may keep birth weight of offspring within the appropriate range. Health education of balanced diet and individual nutrition guidance during pregnancy should be strengthened, to make the dietary structure during pregnancy are more reasonable, reduce the occurrence of adverse birth weight of newborns.

## Introduction

Pregnancy is a complex physiological process, the health of pregnant women and offspring is affected by nutrition during pregnancy. Reasonable and correct dietary structure is the guarantee for improving the quality of the population [1]. The dietary nutrition evaluation of pregnant women was concentrated on the quantitative intake of certain nutrients or specific foods in previous study [2]. The different nutrients contained in each food have intricate interactions in the digestion, absorption and metabolism of the body [3], while the dietary pattern during pregnancy may more fully evaluate the relationship between dietary nutrition and pregnancy outcomes. Dietary pattern is a semi-quantitative research method, but it is suitable for large-scale epidemiological research [4]. In recent years, it has gradually become an important method to explore the dietary nutrition and healthy outcome. The analysis of the overall dietary status can better reflect people’s actual eating conditions and predict the risk of disease [5–7]. For example, Arkkola T [8] analyzed nutritional status during pregnancy, obtained and named 7 dietary patterns in Finland, which largely explained the changes of energy and most nutrients in pregnant women, and the age and education level of pregnant women were correlated with dietary patterns. In addition to reflecting nutrient intake, dietary patterns were also used to analyze associations with pregnancy outcomes. There were few studies on the relationship between dietary patterns of women during pregnancy and neonatal birth weight, and some of the results of these studies were inconsistent. A cohort study from Denmark [9] found that pregnant women in the “Western” diet group were more likely to deliver low birth weight newborns, increasing the risk of small for gestational age (SGA); while pregnant women in the “health-conscious” and “intermediate” groups was associated with a lower incidence of SGA. A survey from the Auckland region of New Zealand found that pregnant women with higher scores on “traditional” dietary patterns had a lower risk of SGA [10]. A study from Norway found that women with a high prudent diet gave birth to babies with significantly lower birth weight and that women with a high traditional diet gave birth to babies with significantly higher birth weight. Moreover, being in the high prudent group was associated with an increased risk of SGA and decreased risk of larger for gestational age (LGA), in comparison with being in the high Western group [11].

However, there are few research data in this aspect in China at present, and the dietary pattern has a strong local specialties and is influenced by many aspects. Chinese pregnant women have complex and diverse eating behaviors, and follow a set of dietary customs which are not extensively explored in the literature. For this reason, a large-scale population survey was conducted in Shaanxi, China. From a macro perspective, the main dietary patterns of women of childbearing age during pregnancy were analyzed, and the possible confounding factors were adjusted to further explore the relationship between dietary patterns and birth weight, SGA and LGA. Information about how maternal food quality is correlated to unfavorable birth weight can provide a basis for nutritional interventions to improve both maternal and infant health in western China.

## Materials and methods

### Sources and subjects

The data were derived from a cross-sectional program named “The prevalence and risk factors of birth defects in Shaanxi Province” ,which was the Sub-topics of National Natural Science Foundation of China project “The primary preventive effect of micronutrient intervention on the occurrence of congenital heart disease during perinatal period and its mechanism”, which were collected from July to November in 2013. A stratified multistage random sampling method was used to carried on sampling, random sampling of districts and counties was carried out after stratification by urban and rural areas. According to the urban-rural ratio and taking into account the population intensity and fertility level, 10 urban districts and 20 counties were randomly selected. The difference between urban and rural areas was determined according to the overall urban and rural population in Shaanxi, making the sample representative. The sampling methods were as follows: 1) 6 townships were randomly selected from the sample counties, and 6 villages were randomly selected from each township. Each village randomly surveyed 30 women aged 15–49 who were pregnant after 2010 and had a clear pregnancy outcome. 2) In each of the urban districts, 3 sub-district offices were randomly selected, and each district randomly selected 6 communities, each community randomly surveyed 60 eligible women of childbearing age. The subjects included women of childbearing age who had been pregnant between January 2010 to November 2013, met the inclusion criteria, had known outcome and informed consent. The inclusion criteria were that women were pregnant from 2010 to 2013, had no major diseases, were willing to participate in this study, and completed the questionnaire independently under the guidance of the investigator. The exclusion criteria were those with twin or multiple pregnancies, with severe memory impairment of dietary intake during pregnancy, and those with diabetes mellitus, severe heart, liver and kidney diseases and psychosis and impairment of understanding ability during gestation. The investigation was approved by the Medical Bioscience Research Ethics Committee of the Ministry of Medicine of Xi’an Jiaotong University (No. 2012008), and all respondents signed informed consent. In order to reduce the error caused by recall bias and increase the accuracy of the results, this study selected children aged between 0 and 1 year old and their mothers with a complete dietary survey data as subjects.

Data collection was conducted using the questionnaire “Status of Birth Defects and Risk Factors in Shaanxi Province”. The content of questionnaire included: basic conditions of the mother, birth weight of the newborn, sociodemographic characteristics (family address, parent’s name, contact information, mother’s gestational age, past birth history, family history, parental age, parental ethnicity, parental marital status, parental residence territory, parental education, parental occupation, family monthly expenditure, mother’s life behavior factors (smoking status, drinking status), etc., food frequency questionnaire (frequency and amount of food consumed by the mother during most recent pregnancy).

### Dietary survey

Food intake was conducted by trained investigators using the semi-quantitative food frequency questionnaire (FFQ) [12]. Considering the consumption and frequency of food in the survey population, this dietary frequency questionnaire included 102 kinds of foods, including 13 kinds of staple foods, 19 kinds of animal protein foods, and 6 kinds of plant protein foods, 36 kinds of vegetables, 12 kinds of fruits, 3 kinds of nut foods, 5 kinds of drinks, and 8 kinds of snacks. After explaining the contents of the questionnaire to the respondents, the investigators uniformly recorded the frequency and amount of each type of food intake of the pregnant woman during the most recent pregnancy. Frequency was divided into: almost don’t eat, < 1 time/month, 1–3 times/month, 1 time/week, 2–4 times/week, 5–6 times/week, 1 time/day, ≥ 2 times/day; each intake was gram per time. This questionnaire was developed based on the food frequency questionnaire of women of childbearing age in Shaanxi Province and verified by a 24-h food review survey. The correlation coefficient between the two methods was 0.6 ~ 0.9, suggesting that the questionnaire had certain reliability and validity [13].

### Newborn Birth Weight (g)

Information about the birth wight of the newborn was collected mainly through birth certificates. When the newborn weight < 2500 g, it was defined as low birth weight infant; when the newborn weight ≥ 4000 g, it was defined as macrosomia; when the newborn birth weight 2500 ~ 4000 g, it was defined as normal weight infant [14]. Referring to the revised report on the birth weight of newborns of different gestational ages in China, the newborns were divided into three categories according to the relationship between birth weight and gestational age: infants were defined as small for gestational age (SGA) when birth weight was below the 10th percentile (P_10_) of the average gestational age; infants with birth weight above the 90th percentile (P_90_) of the same gestational age were defined as larger for gestational age (LGA); between P_10_ and P_90_ of the same gestational age was appropriate for gestational age (AGA) [15].

### Quality Control

Before the implementation of the investigation, a rigorous scientific research design was carried out, and a training manual for investigators was prepared, all investigators received unified training. A community or village was selected for pre-investigation to ensure that each investigator correctly understood each item of the investigation form, and participated in the on-site investigation with a certificate of quality. The completed questionnaires were collated and verified by the investigators. The questionnaires that didn’t answer according to the regulations and had too many missing items were defined as invalid questionnaires, and the invalid questionnaires were excluded. After each county (district) completed the on-site investigation, 5% of the subjects were randomly selected for repeated investigation, and the results were reproducible to ensure the authenticity of the survey results.

### Statistical analysis

The questionnaire data were inputted by Epidata 3.1, and the data was double-input. Due to regional differences in intake of food and nutrient, the analysis was conducted stratified according to women’s location (urban/rural) and region(the southern, northern and middle region). The continuous variables were statistically described by mean ($\overline{x}$) and standard deviation (SD), median (M) and quartile (Q_1_ ~ Q_3_); categorical variables were described by composition ratio and rate. For missing values, we used the mean padding method for processing. These analyses were performed with the use of SAS.9.1, SPSS18.0, Mplus6.0, the drawing process was carried out by Microsoft Office 2003 Excel and Visio software. *P* values (two sided) < 0.05 was considered statistically significant.

#### Factor analysis

In the establishment of the dietary pattern, taking into account the statistical efficiency, the similar foods with particularly small intakes were sorted and combined, and 102 foods were integrated into 31 food items including pasta, rice/porridge, pork, beef and mutton, chicken and duck meat, animals viscera, fish, shrimps and crabs, milk, soy products, vegetables, fruits, fungus, nuts, snacks and drinks. The main dietary patterns were identified according to Principal Component Analysis (PCA) and factor analysis [4]. All 31 food items were integrated into the analysis, the variance was used to maximize the orthogonal rotation to ensure that the factor was clear and the factor load was obtained. The selection of the number of factors was mainly based on: 1) eigen root> 1; 2) the gravel diagram indicated the main factor distribution; 3) interpretability of the extraction factor; 4) the proportion of factors explain variance, but only for reference (because this criterion was largely influenced by the number of variables included in the analysis) [16]. The naming of the factor was based on the characteristics of the food contained in the dietary pattern [17], and the factor was named after the food that best represents the nature of the factor; and according to the characteristics of the factor load, the food with an absolute value of more than 0.3 was selected for analysis.

All participants received factor scores for each dietary pattern, the scores of each dietary pattern were categorized into tertiles (Tertile1 (low score group), Tertile2 (middle score group) and Tertile3 (high score grouping)), the middle tertile (T2) score of each dietary pattern was considered to be the reference. The birth weight of the newborn was used as the dependent variable, and each classified individual dietary pattern was as an independent variable, the logistic regression model was used to investigate the association between birth weight and maternal dietary patterns during pregnancy, and the crude and multivariate-adjusted *OR* and 95%*CI* were calculated. Covariates included infant gender; mother’s age and gestational age, mother’s residence; education level; husband’s residence; family monthly expenditure; regional classification. Further stratified analysis according to the residence (urban, rural) and regional (the southern, northern and middle region).

#### Latent class analysis (LCA)

Firstly, Lo-Mendell-Rubin likelihood ratio test was used to determine the optimal number of LCA selections and compared with dietary patterns after extracting four dietary patterns by factor analysis. The most widely used signal evaluation indicators in the LCA was AIC criterion (akaike information criterion) and the BIC criterion (bayesian information criterion), both indicated that the smaller the value, the better the fit. In the LCA analysis, every women could predict a certain type of probability, and subjects could be grouped into the type of dietary pattern with the highest probability. Then the study mainly used the LCA method to calculate the intake of various foods in each dietary pattern, compared the sociodemographic characteristics and nutrient intake between the various dietary patterns, multiple tests were compared by anova, mann-whitney test and Bonferroni correction.

## Results

### Baseline characteristics of subjects

#### Maternal social demographic characteristics

A total of 15,980 pregnant women who met the requirements were included in the study, aged 17 to 47 years (27.6 ± 4.5). There were 8501 (53.2%) male infants and 7479 (46.8%) female infants; they were aged between 0 to 12 months (6.6 ± 2.6), and the birth weight was 1050-6250 g (3279.9 ± 454.6 g). The basic characteristics of the research object were shown in Table 1.

**Table 1:** Basic characteristics of the respondent

Characteristics	n	result
Mean maternal age, years ($\overline{x}\pm s$)	15,980	27.6 ± 4.5
Family monthly expenditure ( $\yen, \overline{x}\pm s$ )	15,980	2789.6 ± 531.8
Mother’s place of residence (%)		
Urban	3835	24.0
Rural	12,145	76.0
Region (%)		
Southern Shaanxi Region	4395	27.5
Northern Shaanxi Region	2796	17.5
Middle Shaanxi Region	8789	55.0
Educational levels (%)		
Elementary school and below	1326	8.3
Junior high school	7463	46.7
High school and secondary school	3532	22.1
University degree and above	3659	22.9
Occupation (%)		
Peasantry	9940	62.2
Worker	975	6.1
Private teacher	368	2.3
Cadres and civil servants	335	2.1
Business and service industry	1806	11.3
Technicians and other intellectuals	479	3.0
Soldier	2077	13.0
Newborn age (month, $\overline{x}\pm s$)	15,980	6.6 ± 2.6
Gender (%)		
Baby boy	8501	53.2
Baby girl	7479	46.8
Birth weight (g, $\overline{x}\pm s$_)_	15,980	3279.9 ± 454.6

#### Newborn birth weight

In this study, the average weight of newborns in urban areas was 3339.2 ± 461.6 g, 3230.7 ± 451.0 g in rural areas, 3263.7 ± 453.3 g in southern Shaanxi, 3290.3 ± 464.7 g in northern Shaanxi, and 3252.6 ± 450.9 g in middle region. When there was no stratification, 783 newborns with birth weight of < 2500 g, accounting for 4.9%, and 703 newborns with birth weight > 4000 g, accounting for 4.4%; the incidence of SGA and LGA was 14.7 and 7.6%, respectively (Table 2).

**Table 2:** Newborn birth weight distribution (*N* = 15,980)

	$\overline{x}\pm s$	Grouped by birth weight						Grouped by birth weight and gestational age					
		< 2500 g		2500 ~ 4000 g		> 4000 g		SGA		AGA		LGA	
		n	%	n	%	n	%	n	%	n	%	n	%
Area													
Urban	3339.2 ± 461.6	607	3.8	14,542	91.0	831	5.2	1998	12.5	12,464	78.0	1518	9.5
Rural	3230.7 ± 451.0	943	5.9	14,462	90.5	575	3.6	2493	15.6	12,400	77.6	1087	6.8
Region													
Southern Shaanxi Region	3263.7 ± 453.3	783	4.9	14,526	90.9	671	4.2	2397	15.0	12,353	77.3	1230	7.7
Northern Shaanxi Region	3290.3 ± 464.7	943	5.9	14,366	89.9	671	4.2	2525	15.8	12,400	77.6	1055	6.6
Middle Shaanxi Region	3252.6 ± 450.9	703	4.4	14,542	91.0	735	4.6	1966	12.3	12,752	79.8	1262	7.9
Total	3279.9 ± 454.6	783	4.9	14,494	90.7	703	4.4	2349	14.7	12,417	77.7	1214	7.6

### Pregnancy dietary pattern

#### Dietary patterns during pregnancy in women of childbearing age

Factor analysis was used to extract four dietary patterns during pregnancy: “vegetarian pattern”, “balance pattern”, “traditional pattern” and “processing pattern”. Food groups with high factor loadings on dietary pattern 1 included 3 kinds of vegetables, 4 kinds of fruits, pasta, rice/porridge, soy products, fungus mushrooms, it was worth noting that animal protein foods were absent in this model. Food groups with high factor loadings on dietary pattern 2 included 6 kinds of animal protein foods, 3 kinds of vegetables, pasta, rice/porridge, soy products, potatoes, fungus mushrooms, kelp seaweed, melon fruits, nuts, etc., which was characterized by a variety of foods. Dietary pattern 3 was characterized by high factor loadings for rice/porridge, pasta, steamed rice noddles, eggs, milk and dairy, soy products, potatoes, vegetables and fruits, etc. Dietary pattern 4 was characterized by high factor loadings for preserved foods and beverages, snacks, and also included a small amount of vegetables, soy products and staple foods. Among the four dietary structures, the vegetarian pattern was a predominant pattern, which explained the 13.63% of the variability in the predictors, and the latter three patterns explained 9.28, 7.62 and 5.96% of the variability in the predictors, respectively (Table 3).

**Table 3:** Factor Analysis of Dietary Patters of Women of Childbearing Age^a^

Factor 1:Vegetarian Pattern		Factor 2:Balance Pattern		Factor 3:Traditional Pattern		Factor 4:Processing Pattern	
Foods	Factor load	Foods	Factor load	Foods	Factor load	Foods	Factor load
Pasta	0.326	Pasta	0.425	Rice/porridge	0.486	Pasta	0.488
Rice/porridge	0.318	Rice/porridge	0.379	Pasta	0.479	Steamed rice noddles	0.326
Bean products	0.394	Chicken/duck egg	0.323	Steamed rice noddles	0.369	Instant noodles	0.587
Root vegetables	0.551	Chicken/duck	0.338	Potatoes	−0.402	Bacon / bacon / sausage	−0.601
Melon vegetables	0.586	Red meat, beef and mutton	0.326	Mike and dairy	0.326	Bean products	0.319
Green vegetables	0.420	Fish	0.356	Chicken/duck egg	0.349	Root vegetables	0.328
Fungus mushrooms	0.398	Animal’s guts	0.329	Bean products	0.395	Green vegetables	−0.396
Pome fruit	0.652	Mike and dairy	0.498	Pome fruit	−0.645	Snacks	0.556
Citrus fruit	0.388	Bean products	0.486	Citrus fruit	−0.519	Nuts	−0.318
Berry fruit	0.529	Root vegetables	0.571	Root vegetables	0.409	Drink	0.509
Melon fruit	0.765	Melon vegetables	0.401	Green vegetables	0.569		
		Potatoes	0.316	Fungus mushrooms	0.330		
		Green vegetables	0.398				
		Fungus mushrooms	−0.405				
		Kelp seaweed	0.369				
		Melon fruit	0.468				
		Nuts	0.392				
Variance interpretation ratio (%)							
13.63		9.28		7. 62		5.96	

^a^The principal component analysis method was used to extract the factor, and the variance maximization orthogonal method was used to rotate, and the factor load was obtained, and the food item with the absolute value of the retention factor load exceeding 0.3 was obtained

#### Dietary patterns during pregnancy were studied by LCA

**Fig. 1:**
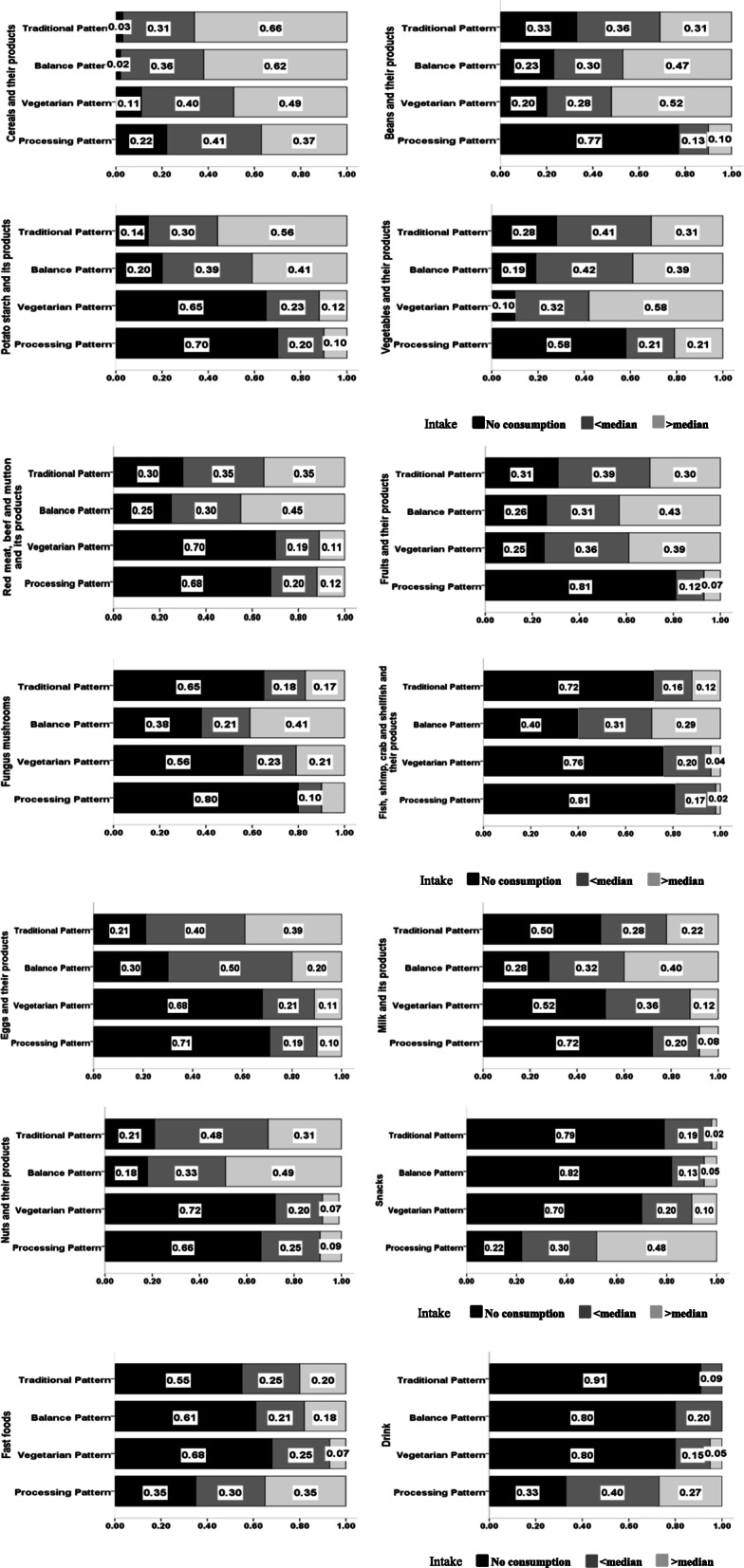
Food intake under various dietary patterns

We chose 4 classes by LCA analysis because at which time the value of AIC (1307.28) and BIC (1311.01) were the smallest, the optimal model was obtained. This result was consistent with the classification of dietary patterns obtained by factor analysis. Figure 1 showed the intake of 14 foods in each dietary pattern (with the median intake as the limit, the group was divided into three groups: greater than, less than the median and no intake). The LCA-Traditional class had higher intake of cereals, potatoes, beans, vegetables, fruits, livestock and poultry, and eggs. The LCA-Balance class had higher intake of cereals, potatoes, beans, vegetables, fruits, fungus mushrooms, livestock and poultry meat, fish, shrimps, crabs, shellfish, milk, and nuts. The LCA-Vegetarian class had higher intake of cereals, beans, vegetables, fruits, livestock and poultry. The LCA-Processing class had higher intake of cereals, vegetables, snacks, instant food, and beverages.

#### Social demographic factors affecting the classification of dietary patterns

In this study, 5881 subjects were included in the vegetarian pattern, 4778 in the balance pattern, 4442 in the traditional pattern, and 879 in the processing pattern. The social demographic characteristics that had an impact on the classification of dietary patterns included family monthly expenditure (*P* = 0.018), the higher the monthly household expenditure, the more inclined the balance pattern and processing pattern, while the lower ones were more likely to choose the vegetarian pattern. Urban women were more inclined to choose balanced pattern, rural women were more inclined to choose vegetarian pattern (*P* = 0.020). The higher the education level of the mother, the more inclined to choose the balanced pattern, the lower the education level, the more inclined to vegetarian pattern and processing pattern(*P* = 0.035). Women with gestational age of less than 37 weeks tended to choose the traditional model, while women with gestational age of more than 37 weeks tended to choose the balanced model (*P* = 0.003). Women in southern region mostly relied on balanced pattern and processing pattern, women in northern region were mostly based on traditional pattern, while women in middle region were mostly vegetarian pattern (*P* < 0.001) (Table 4).

**Table 4:** Social demographic factors affecting the classification of dietary patterns (*N* = 15,980)

Confounders	Vegetarian Pattern(*n* = 5881)	Balance Pattern(*n* = 4778)	Traditional Pattern(*n* = 4442)	Processing Pattern(*n* = 879)	*P*-Value
Mean maternal age, (years, $\overline{x}\pm s$)	27.6 ± 4.8	27.9 ± 4.5	27.5 ± 4.5	26.9 ± 4.3	0.530
Family monthly expenditure (¥, $\overline{x}\pm s$)	2277.4 ± 361.5	2889.6 ± 431.7	2331.2 ± 226.4	2555.3 ± 389.2	**0.018**
Mother’s place of residence (n(%))					
Urban	676(11.5)	1209(25.3)	817(18.4)	200(22.8)	**0.020**
Rural	5205(88.5)	3569(74.7)	3625(81.6)	679(77.2)	
Educational status (n(%))					
Elementary school and below	1594(27.1)	879(18.4)	1133(25.5)	252(28.6)	**0.035**
Junior high school	3564(60.6)	879(18.4)	1577(35.5)	495(56.3)	
High school and secondary school	553(9.4)	2399(50.2)	1355(30.5)	91(10.4)	
University degree and above	170(2.9)	621(13.0)	377(8.5)	41(4.7)	
Gender (n(%))					
Baby boy	3118(53.0)	2551(53.4)	2288(51.5)	470(53.5)	0.488
Baby girl	2763(47.0)	2227(46.6)	2154(48.5)	409(46.5)	
Newborn age (n(%))					
≤ 37 weeks	453(7.7)	134(2.8)	484(10.9)	77(8.8)	**0.003**
> 37 weeks	5428(92.3)	4644(97.2)	3958(89.1)	802(91.2)	
Area (n(%))					
Southern Shaanxi Region	541(9.2)	1410(29.5)	458(10.3)	269(30.6)	**< 0.001**
Northern Shaanxi Region	1588(27.0)	836(17.5)	2021(45.5)	180(20.5)	
Middle Shaanxi Region	752(63.8)	2532(53.0)	1963(44.2)	430(48.9)	

#### Nutrient intake under different dietary patterns

Processing pattern (average energy intake was 2789 ± 853 kcal) and balanced pattern (average energy intake was 2527 ± 895 kcal) compared to traditional pattern (average energy intake was 2395 ± 824 kcal) and vegetarian pattern (average energy intake was 2325 ± 899 kcal), more easy to intake more energy (*P* = 0.005); balanced pattern (average protein intake of 78 ± 9 g) was more adequate than the other three groups of protein intake (*P* < 0.001); processing pattern (average carbohydrate intake was 389 ± 133 g) and traditional pattern (average carbohydrate intake was 377 ± 137 g) compared to the balanced pattern (average carbohydrate intake was 349 ± 132 g) and the vegetarian pattern (average carbohydrate intake was 369 ± 126 g), were more likely to intake more carbohydrate (*P* = 0.040); processing pattern (average cholesterol intake of 220 ± 105 g) was higher than the other three groups of cholesterol intake (*P* < 0.001); balanced pattern (average vitamin A intake was 637 ± 452μgRE) was more adequate than the other three groups of vitamin A intake (*P* = 0.026); balanced pattern (average vitamin B_12_ intake was 2.5 ± 0.5 μg, average folic acid intake was 559 ± 227 μgDFE, average calcium intake was 906 ± 365 mg) was higher than the other three groups of vitamin B_12_, folic acid, calcium intake (*P* < 0.001); the balanced pattern (average iron intake was 33 ± 12 mg) was more adequate than the other three groups of iron intake (*P* = 0.044). For other nutrients, there was no difference between the four dietary patterns (Table 5).

**Table 5:** Mean (standard deviation) of daily absolute nutrient intakes according to clusters of dietary patterns among 15,980 pregnant women ($\overline{x}\pm s$)

	Vegetarian Pattern	Balance Pattern	Traditional Pattern	Processing Pattern	*P*-Value
Energy (Kcal)	2325 ± 899	2527 ± 895	2395 ± 824	2789 ± 853	**0.005**
Protein (g)	60 ± 12	78 ± 9	65 ± 10	58 ± 8	**< 0.001**
Fat (g)	68 ± 9	70 ± 6	69 ± 8	75 ± 10	0.052
Carbohydrate (g)	369 ± 126	349 ± 132	377 ± 137	389 ± 133	**0.040**
Insoluble fiber (g)	33 ± 10	31 ± 11	31 ± 11	28 ± 12	0.088
Cholesterol (g)	50 ± 40	200 ± 100	110 ± 60	220 ± 105	**< 0.001**
Total vitamin A (μgRE)	521 ± 389	637 ± 452	529 ± 353	520 ± 376	**0.026**
Carotene (μg)	2531 ± 2167	2908 ± 2243	2729 ± 2238	2482 ± 2096	0.153
Thiamine (mg)	1.1 ± 0.2	1.2 ± 0.3	1.0 ± 0.2	0.9 ± 0.4	0.189
Riboflavin (mg)	1.2 ± 0.5	1.3 ± 0.4	1.2 ± 0.4	1.2 ± 0.5	0.353
Vitamin B_6_ (mg)	1.5 ± 0.6	1.7 ± 0.5	1.5 ± 0.5	1.3 ± 0.6	0.226
Vitamin B_12_ (μg)	1.2 ± 0.4	2.5 ± 0.5	1.7 ± 0.6	1.0 ± 0.5	**< 0.001**
Folic acid (μgDFE)	293 ± 174	559 ± 227	325 ± 215	278 ± 202	**< 0.001**
Nicotinic acid (mg)	15 ± 7	16 ± 9	16 ± 7	15 ± 7	0.338
Vitamin C (mg)	98 ± 64	109 ± 75	97 ± 67	95 ± 63	0.067
Vitamin E (mg)	10 ± 3	11 ± 4	10 ± 4	9 ± 3	0.157
Calcium (mg)	614 ± 335	906 ± 365	758 ± 352	622 ± 351	**< 0.001**
Phosphorus(mg)	942 ± 554	1082 ± 600	995 ± 533	1210 ± 613	0.088
Potassium (mg)	2413 ± 121	2557 ± 140	2535 ± 128	2665 ± 146	0.076
Sodium(mg)	2502 ± 212	2659 ± 235	2889 ± 253	3003 ± 267	0.055
Magnesium(mg)	405 ± 193	432 ± 202	423 ± 198	402 ± 189	0.565
Iron (mg)	22 ± 10	33 ± 12	25 ± 11	20 ± 10	**0.044**
Zinc (mg)	6.5 ± 4.1	7.6 ± 4.4	6.9 ± 3.9	6.2 ± 3.8	0.259
Selenium(mg)	34 ± 25	40 ± 28	36 ± 26	32 ± 21	0.287
Copper(mg)	2.0 ± 1.0	2.2 ± 1.2	2.1 ± 1.1	2.2 ± 1.1	0.474
Iodine(mg)	211 ± 98	228 ± 108	220 ± 101	225 ± 112	0.293

### The association between dietary patterns during pregnancy and neonatal birth weight

Unstratified analysis, women in the lowest tertile of adherence to vegetarian pattern had lower risk of low birth weight in offspring compared with moderate tertile (*OR* = 0.67, 95%*CI*: 0.46–0.98), women in the highest tertile increased the risk of low birth weight in offspring (*OR* = 1.82, 95%*CI*: 1.36–3.77), and the results remained unchanged after adjusting for covariates. In rural areas, vegetarian pattern was positively associated with a higher risk of low birth weight (*OR* = 1.61, 95%*CI*: 1.06–2.93), the processing pattern was found a protective factor for the occurrence of low birth weight (*OR* = 0.98, 95%*CI*: 0.43–0.99). The same results were not shown in urban areas.

After stratification by region, compared with the moderate processing pattern, T_1_ and T_3_ groups in pregnant women could increase the risk of low birth weight in offspring in southern Shaanxi region, the T_3_ group was statistically significant (*OR* = 8.83, 95%*CI*: 1.22–15.16). In northern Shaanxi region, the balanced pattern T_1_ group could increase the risk of low birth weight (*OR* = 1.35, 95%*CI*: 1.14–3.85), the T_3_ group reduced the risk of low birth weight (*OR* = 0.35, 95%*CI*: 0.14–0.83). In middle region, women in the highest tertile of adherence to vegetarian pattern had higher risk of low birth weight in offspring (*OR* = 1.75, 95%*CI*: 1.18–2.62). The traditional pattern T_1_ group could reduce the risk of low birth weight in offspring (*OR* = 0.80, 95%*CI*: 0.39–0.93), the T_3_ group increased the risk of low birth weight (*OR* = 1.55, 95%*CI*: 1.05–3.75) (Table 6).

**Table 6:** The relationship between four dietary patterns and birth weight

	Urban or Rural				Area						Total	
	Urban		Rural		Southern Shaanxi Province		Northern Shaanxi Province		Guanzhong Shaanxi Province			
	OR	OR’	OR	OR’	OR	OR’	OR	OR’	OR	OR’	OR	OR’
	(95%CI)	(95%CI)	(95%CI)	(95%CI)	(95%CI)	(95%CI)	(95%CI)	(95%CI)	(95%CI)	(95%CI)	(95%CI)	(95%CI)
Vegetarian Pattern												
Tertile 2	1.00	1.00	1.00	1.00	1.00	1.00	1.00	1.00	1.00	1.00	1.00	1.00
Tertile 1	0.75	0.66	0.54*	0.46*	1.50	1.42	0.61	0.58	0.91	0.82	0.67*	0.56*
	(0.53–2.32)	(0.37–2.06)	**(0.38–0.91)**	**(0.29–0.88)**	(0.37–6.18)	(0.32–6.09)	(0.28–1.33)	(0.25–1.28)	(0.64–1.28)	(0.36–1.62)	**(0.46–0.98)**	**(0.41–0.83)**
Tertile 3	1.72	2.16	1.61*	2.08*	0.81	0.68	2.47	2.59	1.75*	1.89*	1.82*	2.32*
	(0.66–3.32)	(0.73–4.28)	**(1.06–2.93)**	**(1.48–4.83)**	(0.27–2.40)	(0.22–2.36)	(0.67–9.11)	(0.72–9.01)	**(1.18–2.62)**	**(1.23–3.05)**	**(1.36–3.77)**	**(1.59–3.89)**
Balance Pattern												
Tertile 2	1.00	1.00	1.00	1.00	1.00	1.00	1.00	1.00	1.00	1.00	1.00	1.00
Tertile 1	1.54	1.46	1.28	1.39	1.48	1.27	1.35*	1.22*	1.83	1.51	2.63	2.86
	(0.52–3.22)	(0.43–3.06)	(0.70–1.59)	(0.82–1.76)	(0.44–4.94)	(0.36–4.77)	**(1.14–3.85)**	**(1.09–3.55)**	(0.56–3.68)	(0.46–3.53)	(0.66–4.32)	(0.73–4.46)
Tertile 3	0.59	0.38	0.80	0.72	1.26	1.33	0.35*	0.26*	0.85	0.78	0.72	0.58
	(0.24–1.69)	(0.19–1.53)	(0.61–2.68)	(0.48–2.48)	(0.30–5.22)	(0.38–5.89)	**(0.14–0.83)**	**(0.09–0.76)**	(0.51–1.95)	(0.46–2.16)	(0.44–1.76)	(0.39–1.57)
Traditional Pattern												
Tertile 2	1.00	1.00	1.00	1.00	1.00	1.00	1.00	1.00	1.00	1.00	1.00	1.00
Tertile 1	1.36	1.73	0.80	0.67	1.63	1.82	0.82	0.76	0.80*	0.76*	0.88	0.73
	(0.42–2.91)	(0.59–3.28)	(0.43–1.92)	(0.38–1.86)	(0.61–2.95)	(0.55–3.39)	(0.42–3.55)	(0.38–3.26)	**(0.39–0.93)**	**(0.32–0.86)**	(0.58–2.91)	(0.49–2.67)
Tertile 3	0.98	0.83	1.66	2.02	1.15	1.26	1.72	1.98	1.55*	1.76*	1.35	1.38
	(0.65–2.12)	(0.53–2.01)	(0.39–4.72)	(0.58–5.01)	(0.58–3.71)	(0.61–3.66)	(0.63–3.97)	(0.78–4.06)	**(1.05–3.75)**	**(1.12–3.98)**	(0.79–3.12)	(0.76–3.31)
Processing Pattern												
Tertile 2	1.00	1.00	1.00	1.00	1.00	1.00	1.00	1.00	1.00	1.00	1.00	1.00
Tertile 1	0.63	0.55	1.23*	1.39*	6.57	0.76	1.50	1.52	1.67	1.76	1.68	1.45
	(0.20–1.97)	(0.18–1.88)	**(1.06–2.86)**	**(1.11–3.04)**	(0.81–13.82)	(0.42–15.48)	(0.67–3.36)	(0.53–3.83)	(0.39–3.08)	(0.42–3.16)	(0.59–3.53)	(0.51–3.48)
Tertile 3	0.80	0.72	0.98*	0.83*	8.83*	8.97*	0.70	0.82	0.86	0.75	0.63	0.72
	(0.40–1.62)	(0.33–1.85)	**(0.43–0.99)**	**(0.40–0.92)**	**(1.22–15.16)**	**(1.36–16.34)**	(0.28–1.74)	(0.49–2.26)	(0.29–1.56)	(0.22–1.42)	(0.40–1.62)	(0.53–1.88)

Tertile 1, 2, and 3 refer to the lower tertile, middle tertile, and upper tertile of the dietary pattern factor score, respectively, with Tertile 2 as the reference group. OR and OR’ are risk estimates before and after adjustment of covariates, including infant gender, gestational age, mother’s age, mother’s place of residence (urban and rural), maternal education, husband’s place of residence (urban and rural), family monthly expenditure, region classification (southern region, northern region, middle region)

*OR* Odds ratios, *CI* Confidence interval

*P < 0.05

## Discussion

The incidence of birth defects and low birth weight was high in Shaanxi, China [18], but there was a lack of effective evaluation of the dietary patterns that dominated local women of childbearing age during pregnancy and their impact on newborn birth weight. This study evaluated the effects of different dietary patterns on neonatal birth weight, and provided a theoretical and practical reference for dietary nutrition interventions to improve women’s dietary patterns during pregnancy and reduce abnormal birth weight in Shaanxi Province, China.

At present, there are few studies on the dietary patterns of women of childbearing age during pregnancy, especially in China, where there are hardly any studies on the relationship between dietary patterns and newborn birth weight. In a cohort study in Denmark, the “Mediterranean pattern” reduced the risk of preterm birth; the “Western pattern” was positively associated with the occurrence of forearm fractures, low birth weight, and SGA in the offspring [19]. In this study, four dietary patterns were extracted by factor analysis: “vegetarian pattern”, “balance pattern”, “traditional pattern” and “processing pattern”. The vegetarian pattern was a predominant pattern, characterised by a high intake of vegetables, fruits, soy products and staple foods, with a limited range of variety of foods and a lack of meat and eggs and dairy foods. Long-term use of this kind of dietary pattren could lead to insufficient intake of protein, especially high-quality protein and fat, especially essential fatty acids and vitamin A, calcium, iron, zinc, etc., which caused a series of health problems for mothers and their offspring. The balanced pattern was considered to be reasonable in food mix and suitable for various nutrients. It was recommended that pregnant women should adhere to this dietary pattern for a long time.

Some nutritional epidemiological studies had evaluated the relationship between dietary patterns and sociodemographic characteristics of the population [20–23]. On the basis of factor analysis, this study used LCA to analyze the social demography factors affecting various dietary patterns. The study found that the higher the monthly household expenditure, the more inclined to choose the balance pattern and processing pattern, while the lower ones were more likely to choose the vegetarian pattern; the higher the education level of the mother, the more inclined to choose the balanced pattern, the lower the education level, the more inclined to vegetarian pattern and processing pattern. A Norwegian study on dietary patterns in pregnant women also found that people with higher levels of education and household income tend to choose a balanced pattern rich in vegetables, fruits, nuts, milk, fish and whole grains [24]. Similar results were obtained in several other studies [25–27]. For the special group of pregnant women, in the traditional Chinese concept, people should ate more eggs and meat to supplement nutrition during pregnancy, and those with higher education level were often more health-conscious and had the financial ability to bear foods rich in nutrients [5]. The group with higher education level and economic income level was more inclined to adopt a balanced pattern.Urban women were more inclined to choose balanced pattern, rural women were more inclined to choose vegetarian pattern. A survey of dietary patterns of women in nine provinces in China found that women in rural areas and with low education levels were more likely to choose the potato or vegetarian pattern [28]. This might be related to the wide range of vegetable and potato food sources in rural areas and relatively economically.Women with gestational age of less than 37 weeks tended to choose the traditional model, while women with gestational age of more than 37 weeks tended to choose the balanced model. This was also consistent with the results of relevant reports that pregnant women with traditional dietary patterns were more prone to preterm birth [29]. Women in southern region mostly relied on balanced pattern and processing pattern, women in northern region were mostly based on traditional pattern, while women in middle region were mostly vegetarian pattern, which might be related to the fact that dietary pattern selection was population specific and susceptible to the influence of social culture and food supply [30–32]. The intake of nutrients varied among various dietary patterns, mainly due to the different types of foods contained in various dietary patterns.

This study found that in the unstratified and stratified rural areas and middle region, the incidence of low birth weight of newborns was positively correlated with the score of vegetarian-pattern diet, and the pregnant women with high scores in the traditional pattern also had an increased risk of low birth weight in middle region. Women who adopted these two dietary patterns were prone to cause insufficient intake of energy, protein, essential fatty acids, fat-soluble vitamins, and minerals rich in animal foods such as iron, calcium, and zinc, resulting in fetal growth disorders and affecting normal organ and tissue functions. However, a study in New Zealand showed that the higher the scores of traditional patterns (mainly fruits, vegetables, yogurt and lean meat) in early pregnancy, the lower the incidence of low birth weight [33], possibly due to the fact that our study contains different foods from the traditional patterns defined in this paper. In addition, the processing pattern was found a protective factor for the occurrence of low birth weight in rural areas, but women with high scores in the processing pattern had an increased risk of low birth weight in southern region. This dietary pattern had very few types of food, it was not only lack of foods rich in quality protein such as poultry, fish, shrimps and eggs, but also the staple food was only pasta, the vegetables and fruits were ingested less, and the pickled foods and snacks and drinks were more ingested. So not only lack of nutrients such as protein and unsaturated fatty acids, but also a variety of vitamins, such as vitamin A, B vitamins, vitamin E, vitamin C, etc., and increased the intake of a variety of harmful substances. It was not only unable to meet the nutritional needs of pregnant women, but also far from meeting the needs of fetal growth and development as well as the formation of tissues and organs, which leaded to the stop of fetal intrauterine development, the occurrence of low birth weight, and even premature separation of fetus and placenta [34], leading to reduced survival rate, spontaneous abortion and stillbirth. In rural areas, the reason for the opposite result might be that the sources of snacks, sweet foods, fast foods, and processed foods were few and not easy to obtain, women had eaten such foods during pregnancy, but the amount of consumption was small, such foods contained a lot of calories, so they could increase the birth weight of newborns. The high-scoring balanced pattern was a protective factor for the occurrence of low birth weight in northern areas. Under this dietary pattern, pregnant women were less likely to suffer from malnutrition, and their dietary intake could meet the nutritional needs of the mother and the fetus, and could reduce the occurrence of various adverse pregnancy outcomes, including abnormal birth weight of newborns [35]. Studies by Knudsen [36] had also shown that healthy dietary patterns rich in fruits, vegetables, and meat could significantly reduce the risk of SGA compared to western dietary patterns (high-fat milk, refined grains, and processed meat). A large sample study in Norway (*n* = 66,000) showed that pregnant women who adhered to a prudent diet during pregnancy (mainly fruits, vegetables, vegetable oils and whole grains) had a lower risk of preterm labor than other pregnant women [24]. Although the dietary patterns that favor birth outcomes varied from study to study, they were also consistent that eating low-processed foods such as fruits and vegetables, low-fat milk and lean meat during pregnancy might reduce the risk of low birth weight [37–39].

In summary, the dietary pattern of women of childbearing age may be closely related to the birth weight of the offspring. In order to reduce the occurrence of abnormal birth weight, women should adhere balance pattern during pregnancy, increasing the intake of high quality protein food, especially milk and seafood. It is suggested that dietary intervention during pregnancy should be improved, health education for dietary nutrition should be strengthened, and women should be able to make reasonable meals according to the actual local food supply characteristics. The advantage of this research is that the survey has undergone rigorous scientific design, the quality of the investigators is high, and the on-site work is organized and planned, the collected data is relatively accurate. The sample size of this study is large, and the possible confounding factors are controlled as much as possible during the analysis, increasing the reliability of the conclusion. However, there are still some limitations in this study. First of all, this study mainly adopts the cross-sectional research method, which cannot verify the causal relationship. Secondly, in this study, dietary information during pregnancy was recalled by subjects and collected through FFQ, which suggests that the recall bias could not be avoided. Thirdly, it is subjective to define the dietary pattern according to the concentration of food types contained in the dietary pattern, but the FFQ survey still has important reference value for the study of the dietary structure and food types of the population, especially the factor analysis plays a good role in the classification of dietary pattern [40–43]. Finally, some potential confounding factors may not be controlled during the analysis. This study used dietary patterns to estimate the effect on neonatal birth weight based on population level, providing important research clues for the relationship between dietary patterns of pregnant women and neonatal birth weight, and expanding new ideas. The results of the study will have important reference significance for pre-pregnancy counseling and prenatal care.

## Conclusion

There are four kinds of dietary patterns in the pregnant women of childbearing age in Shaanxi Province, “vegetarian pattern” is predominant, and the dietary structure needs to be improved. The vegetarian and traditional pattern may be positively related to a higher risk of low birth weight while the balanced pattern may keep birth weight of offspring within the appropriate range. Health education of balanced diet and individual nutrition guidance during pregnancy should be strengthened, in order to make dietary structure during pregnancy are more reasonable, reduce the occurrence of adverse birth weight of newborns.

## Data Availability

The data sets supporting the results of this article are included within the article and its additional files.
