# Correction to: Dietary patterns of Chinese women of childbearing age during pregnancy and their relationship to the neonatal birth weight

**DOI:** 10.1186/s12937-020-00637-6

**Published:** 2020-11-03

**Authors:** Hui Yan, Shaonong Dang, Yaodong Zhang, Shuying Luo

**Affiliations:** 1 The Children’s Hospital Affiliaten to Zhengzhou University; Henan Children’s Hospital, Zhengzhou Children’s Hospital, Zhengzhou, 450018 China; 2 School of Public Health, Xi’an Jiaotong University, Xi’an, 710061 China

**Correction to: Nutr J 19:89 (2020)**

**https://doi.org/10.1186/s12937-020-00607-y**

Following publication of the original article [1], the authors missed to indicate Shaonong Dang as a co-corresponding author.

This has been corrected above.
